# Can we prevent social identity switches? An experimental–computational investigation

**DOI:** 10.1111/bjso.12647

**Published:** 2023-04-11

**Authors:** Anna K. Zinn, Miriam Koschate, Elahe Naserianhanzaei, Aureliu Lavric

**Affiliations:** ^1^ Department of Psychology University of Exeter Exeter UK; ^2^ UQ Business School The University of Queensland Brisbane Queensland Australia; ^3^ Institute for Data Science and AI University of Exeter Exeter UK; ^4^ Department of Political Sciences University of Exeter Exeter UK

**Keywords:** cognitive control, identity salience, identity switches, linguistic style, multiple identities, social identity

## Abstract

Previous studies suggested that social identity switches are rapid and highly effective, raising the question of whether people can intentionally control such switches. In two studies, we tested if participants could exert top‐down control to prevent a social identity switch triggered by the experimental context. In Study 1, participants (*N* = 198) were given a writing task aimed at prompting a switch from their parent identity to their feminist identity. Before the prompt, half of the participants (the experimental group) were instructed to remain in their parent identity, avoiding an identity switch; the control group was not given such instructions. We found no significant difference between the groups in either self‐reported salience or the implicit computational measure of salience based on participants' linguistic style, both measures suggesting a switch in both groups. Study 2 (*N* = 380) followed the same design but included a monetary incentive to prevent the switch in the experimental group. The groups differed significantly in their self‐reported salience but not in the implicit measure, which suggests limited ability to avoid the switch even when participants report being able to do so. These results point to limited intentional control over exogenously triggered identity switches, with important practical implications.

## INTRODUCTION

Most people are part of multiple groups – these social identities are thought to define who we are (Tajfel & Turner, 1979). They may include one's social identity as a member of a work team, as a parent or as a supporter of specific beliefs and/or values (e.g. being a feminist). *Social identity switching* – the change to another social identity that becomes more salient – has received surprisingly little scientific scrutiny. The current studies attempt to answer the following key question: can people prevent an identity switch triggered by the social context? This may have practical implications for situations where one might need to maintain the salience of an (e.g. occupational) identity, despite exogenous triggers to switch away from it.

Switching social identities can help us ‘tune into an appropriate view of ourselves in order to behave in a coherent and situationally appropriate manner’ (Hugenberg & Bodenhausen, 2004, p. 234). Van Bavel and Packer (2021) state that: ‘Just as people can switch from sunglasses to regular glasses when they enter a dark building (…), they can switch identities as they move from one situation to the next’. (p. 42). These statements contain two common assumptions about social identity switching: (1) identity switches are rapid and effective when required by circumstances, and (2) people have at least some intentional control over identity switches (as they do over switching glasses).

The first assumption – that switching is rapid and effective – has been empirically tested in recent studies by Zinn et al. (2022). They asked whether social identity switches lead to a delay in activating the switched‐to identity – an ‘identity activation cost’. Using an experimental design loosely modelled on the task‐switching paradigm (Meiran, 1996; Rogers & Monsell, 1995), they compared switching identities to staying in the same identity. Identity salience was inferred from reaction time and error rate patterns in identity‐related Implicit Association Tests (IATs; Greenwald et al., 1998). Each participant completed IATs that prompted an identity switch as well as IATs that prompted the same identity as the preceding IAT. The key finding was that an identity switch did not result in an identity activation cost (measured as reduced identity salience) compared to repeating an identity. This result supports the first assumption above – that social identity switches are effective. However, to the best of our knowledge, the second assumption – that exogenously triggered identity switches are subject to top‐down control – has not been empirically tested. The current studies will attempt such a test.

The salient identity is likely to influence people's perception and behaviour (Chakravarty & Fonseca, 2017; Hackel et al., 2018). Hence, there may be situations in which the social identity prompted by the environment/context is maladaptive. Treating a child of a similar age to the doctors' own might prompt a switch towards the parent identity, potentially affecting the doctor's affective state and decision‐making capacity. Similarly, a judge deciding on an important verdict must avoid switching away from their professional identity. This is especially important as emotions towards other people can be influenced by the currently salient social identity (Kuppens & Yzerbyt, 2012). Therefore, it can be beneficial to have the capacity to prevent a switch to another identity.

### Top‐down control of social identity switches

To learn more about the role of top‐down (intentional) control in identity switching, we draw on research on task switching and language switching (Declerck & Philipp, 2015; Graham & Lavric, 2021; Kiesel et al., 2010; Lavric et al., 2008; Monsell, 2003, 2015). Optimal performance requires a balance of flexibility (to rapidly change tasks) and stability to deal with distraction from, or interference to, the task they are currently completing (Dreisbach & Fröber, 2019). This balance is also likely to be important for social identity switches. It can be argued that a certain level of flexibility helps people to rapidly adapt to different situations by making the most relevant identity salient. On the other hand, having a degree of control over social identity switches is needed for preventing maladaptive exogenously triggered identity switches.

A task‐switching study by Elchlepp et al. (2015) showed that even fast and relatively automatic processes such as word recognition are not beyond people's intentional top‐down control. They found that EEG‐derived brain potentials linked to lexical processing were substantially attenuated, delayed if participants performed a task for which lexical processing was irrelevant (judging the symmetry of coloured letters within a word). This indicates that participants could use top‐down control to allocate (at least to an extent) their attention away from a very highly practiced task such as word reading when another task was required. Graham and Lavric (2021) compared participants' top‐down control of task selection versus language selection. Preparation reduced the switch cost in both domains, but the reduction was much steeper for task switching than language switching, suggesting more effective top‐down control of task selection than language selection (see also Lavric et al., 2019). This shows that there is at least a degree of top‐down control even in processes commonly thought to be automatic, but that its effectiveness varies considerably over domains.

To our knowledge, there has been no research on control of exogenously elicited switches in social identity. The two studies presented here will attempt to determine if an exogenously cued social identity switch can be prevented intentionally.

### Current studies

Our aim is to assess whether participants can stay in a specific identity (their parent identity) when presented with prompts that would be expected to elicit a switch towards another identity (their feminist identity). One strong prompt that may lead to an identity switch is the topic that one thinks/talks/writes about. Hence, we used the writing topic as a prompt to switch away from the parent to the feminist identity. The two social identities (parent and feminist) and the feminist writing topic (objectification of women) were selected based on research by Koschate et al. (2021) who developed a machine‐learning linguistic analysis technique we employ here (see below). We asked whether people in the experimental group could prevent the switch prompted by the writing topic and stay in their parental identity.

In the presented studies, identity salience was assessed explicitly, through a self‐report measure, and inferred implicitly. Self‐report is easy to implement and is one of the most widely used types of methods for assessing identity salience (Abdelal et al., 2009). However, this measure relies heavily on the participant's level of introspection regarding small and possibly automatic changes in salience (cf. Koschate et al., 2021). Hence, our primary measure was the Automated Social Identity Assessment (ASIA; Koschate et al., 2021), which we expected to capture identity salience more objectively. ASIA uses a machine‐learning‐based analysis of linguistic style to assess identity salience. Koschate et al. (2021) trained and tested a computational linguistic classifier model to distinguish between two social identities: parent identity versus feminist identity. The classifier was trained on over 600,000 forum posts from parent and feminist forums. It was then cross‐validated on posts written by forum users who had posted on both a parent and a feminist forum (similar to a within‐subject design) as well as in an experimental design in which salience was manipulated while keeping topic and audience constant. Koschate et al. (2021) found that the style‐based classifier could distinguish accurately between the parent and feminist identities, indicating that people showed distinct linguistic styles consistent with their salient identity (parent vs. feminist). Importantly, rather than analysing *what* people write about (i.e. the content), ASIA focuses on *how* people write (i.e. the style they use) using features such as emotion words, pronouns and the length of words. Importantly, Koschate et al. (2021) show that the accuracy of the classifier is not affected by the topic, even when the topic is more typical of one identity than another. In an experiment, they manipulated the salience of either a parent or feminist identity of participants who consider themselves to be both, a feminist and a parent. Participants were asked to write short texts about three topics: one that a pre‐study had shown to be more typical of parents than feminists, one that was more typical of feminists than parents and one that was untypical of either. The ASIA measure was able to classify participants correctly to the salience condition at very similar levels of accuracy across all three topics. The most frequent words of the most predictive features were words that can be used widely across different topics such as ‘that’, ‘because’, ‘they’, ‘think’, ‘when’ and ‘like’ (see Koschate et al., 2021, Supporting Information). This means that even though the topic overall moves the mean towards one identity (e.g. feminist identity when participants write about objectification of women), the classifier can still distinguish between the two groups.

Our studies build on the findings by Koschate et al. (2021) by investigating whether an overall switch to the identity related to the writing topic (as shown in the mean levels of salience) can be prevented. We compared participants instructed to prevent the exogenously cued switch to the feminist identity versus participants who were not instructed to prevent the switch in their linguistic styles and self‐report. We used the relative salience of the parent and feminist identities as an index of participants' capacity to prevent the switch elicited by the text writing.

Given the evidence from task switching that even highly practiced (habitual) processes (e.g. reading) are subject to top‐down control (Elchlepp et al., 2015), one might expect that social identity switches are also subject to top‐down control, leading to the following hypotheses:

H1 – implicit measure: If people can prevent switching identities, we would expect to find a significant difference between control and experimental condition in their linguistic style with the control condition showing a more feminist style and the experimental condition showing a more parental linguistic style.
H2 – explicit measure: If people can prevent switching identities, there will be a smaller reduction in the self‐reported salience of the parental identity (from directly after the three things manipulation to directly after the writing task) in the experimental condition compared to the control condition.^1^

## STUDY 1 – PREVENTING A SOCIAL IDENTITY SWITCH

### Method

#### Participants

The target sample size was a minimum of 176 participants (see Appendix A).^2^ Participants had to be native English speakers, aged ≥18, who self‐identify as feminists and parents. Participants were recruited in the United Kingdom and Australia through social media (e.g. Facebook, Mumsnet and Netmums) and on Prolific. Participants on Prolific were paid £2.50, whereas social media participants could enter a draw for two £50/AUD100 Amazon vouchers.

Participants who wrote less than 25 words (Koschate et al., 2021) or dropped out before completing the second salience self‐report measure (immediately after the writing task) were excluded from data analyses. The final *N* was 185 (control group: *n* = 92; experimental group: *n* = 93). Participants' mean age was 42.06 years (*SD* = 10.53, min = 22, max = 74); 84.9% identified as female (15.1% male); 81.6% were British and 14.1% Australian (4.3% other nationalities).^3^ The study received approval from the departmental ethics committee at the University of Exeter.

#### Design

The dependent variables were the implicit (linguistic style) and explicit (self‐report) measures of the currently salient social identity. For the implicit measure, which was recorded only at *T*2 (after the identity switch), the study followed a between‐subject design with condition as a two‐level independent variable: the experimental group received a prompt asking them to try to remain in their parent identity, whereas the control group received a neutral prompt. The self‐report measure was acquired both before the switch (*T*1) and after the switch (*T*2), resulting in an extra (within‐participant) independent variable, − time (*T*1 vs. *T*2), in a mixed condition × time design.

#### Materials and procedure

The study was run online using Qualtrics, with participants randomly assigned to the experimental or control condition.

##### Parent identity salience

After proving informed consent, all participants completed a ‘three things’ manipulation (Haslam et al., 1999), aimed at increasing the salience of their parental social identity by asking them to list up to three things that they and other parents do well, badly, often and rarely.

Participants then had to answer the question ‘How strongly do you think of yourself as a parent right now?’ (based on Verkuyten & Hagendoorn, 1998) on a 6‐point Likert scale ranging from 1 (not at all) to 6 (very strongly).

##### Switch prevention manipulation

**FIGURE 1:**
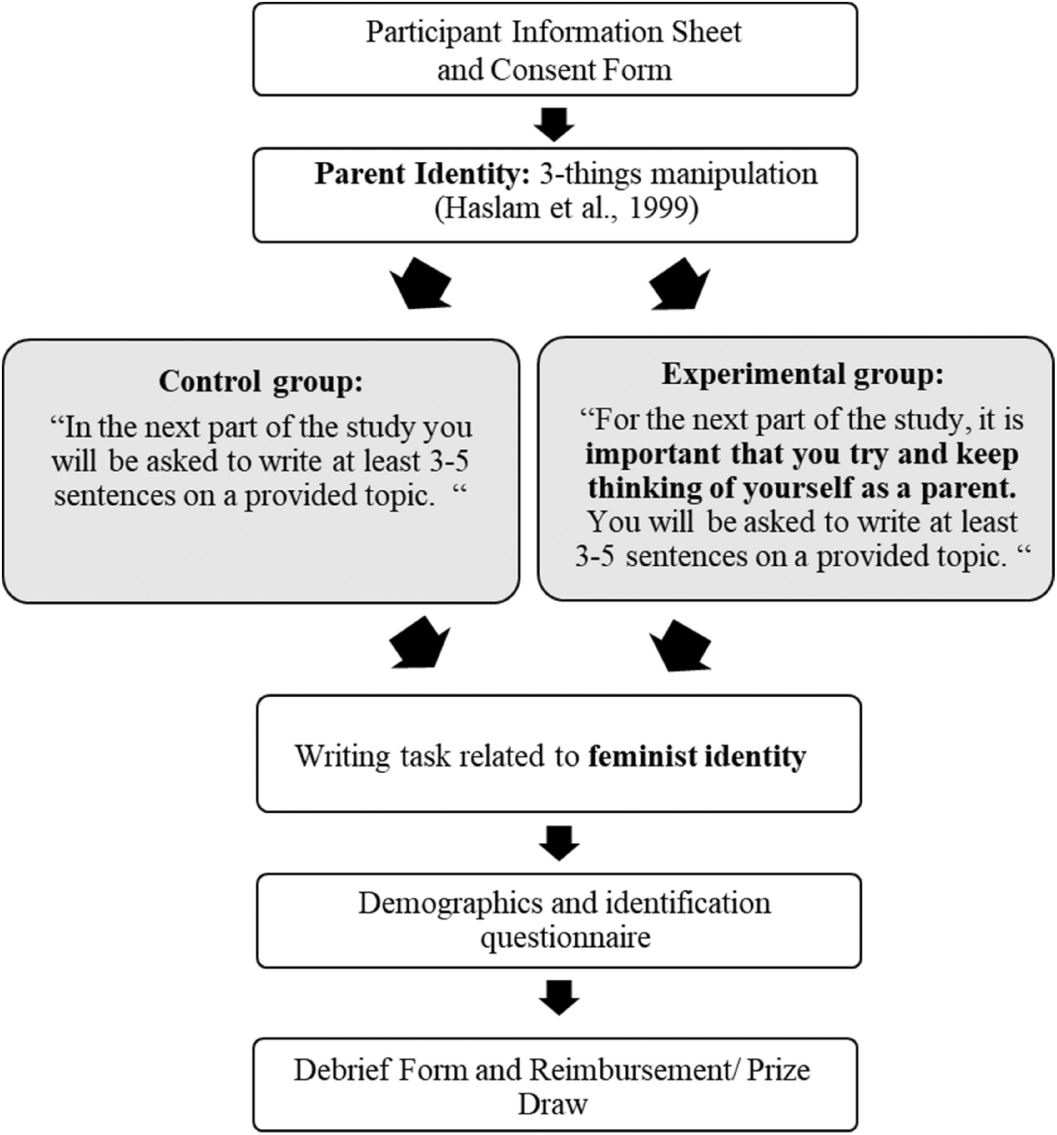
Study 1 Procedure. *Note*: Participants were randomly allocated to the control or experimental group.

As shown in Figure 1, participants in both groups were told: ‘In the next part of the study, you will be asked to write at least 3–5 sentences (25 words) on a provided topic’. Participants in the experimental group were provided with the extra instruction to ‘…try and keep thinking of yourself as a parent’ in the next part of the study.

##### Feminist identity salience

Participants were then asked to write about a topic strongly related to a feminist social identity: ‘Women are still treated like sex objects’. This topic was chosen based on research by Koschate et al. (2021), who found that the topic ‘objectification of women’ is associated significantly more strongly with a feminist identity than with a parent identity.

##### Implicit salience measure

Identity salience during the writing task was assessed through participants' linguistic style using ASIA (Koschate et al., 2021). The linguistic style in the texts written by participants was converted to 41 stylistic indicators using LIWC (Pennebaker et al., 2007). Subsequently, the probability of the parent identity or the feminist identity being salient was calculated using the parent/feminist salience classifier model trained and validated by Koschate et al. (2021). Importantly, ASIA generates a single measure (probability) of the parent versus feminist identity being salient, rather than two separate measures. This means that the salience of an identity is always estimated relative to the salience of the other identity included in the assessment. In line with recommendations by Koschate et al. (2021), domain adaptation (Fernando et al., 2013) was used to adapt the classifier to account for changes to the feature distributions caused by different requirements between the training dataset (online forum posts) and the test dataset (experimental data). For example, we asked participants to write three to five sentences in the experiment which is likely to affect the frequency of words being used irrespective of the salient identities being assessed (see Appendix B).

##### Explicit salience measure

Immediately after the main writing task, participants were asked to answer again the same question that was asked after the three things task. This provided the second (*T*2) measure, allowing the comparison between the self‐reported salience before (*T*1) and after (*T*2) the switch prevention manipulation.

##### Demographics and identification questionnaire

After the second self‐report measure of salience was collected, participants were asked further questions about their identities as parents and feminists, as well as demographic questions (see Supporting Information: S1, for full questionnaire; Appendix C for a summary of measures and sample descriptives^4^). At the end of the study, participants were debriefed and those recruited via Prolific were reimbursed.

### Results

#### Preliminary analysis

##### Randomization checks

Chi‐square tests of independence showed that there was no significant association between the participant's condition (experimental vs. control) and their sex, *χ* ^2^(1, *N* = 185) = 0.62, *p* = .430; male vs. female or whether children were still living at home, *χ* ^2^(1, *N* = 184) = 0.003, *p* = .954. Independent samples *t*‐tests showed no significant difference between the two groups in: age, *t*(182) = −1.03, *p* = .305, *d* = 0.15; age of the youngest child, *t*(181) = −0.86, *p* = .393, *d* = 0.13; count of responses to the three things manipulation, *t*(183) = 0.46, *p* = .645, *d* = 0.07; and word count in main writing task, *t*(183) = 1.00, *p* = .319, *d* = 0.15.

#### Hypothesis testing

##### H1 – implicit measure

**FIGURE 2:**
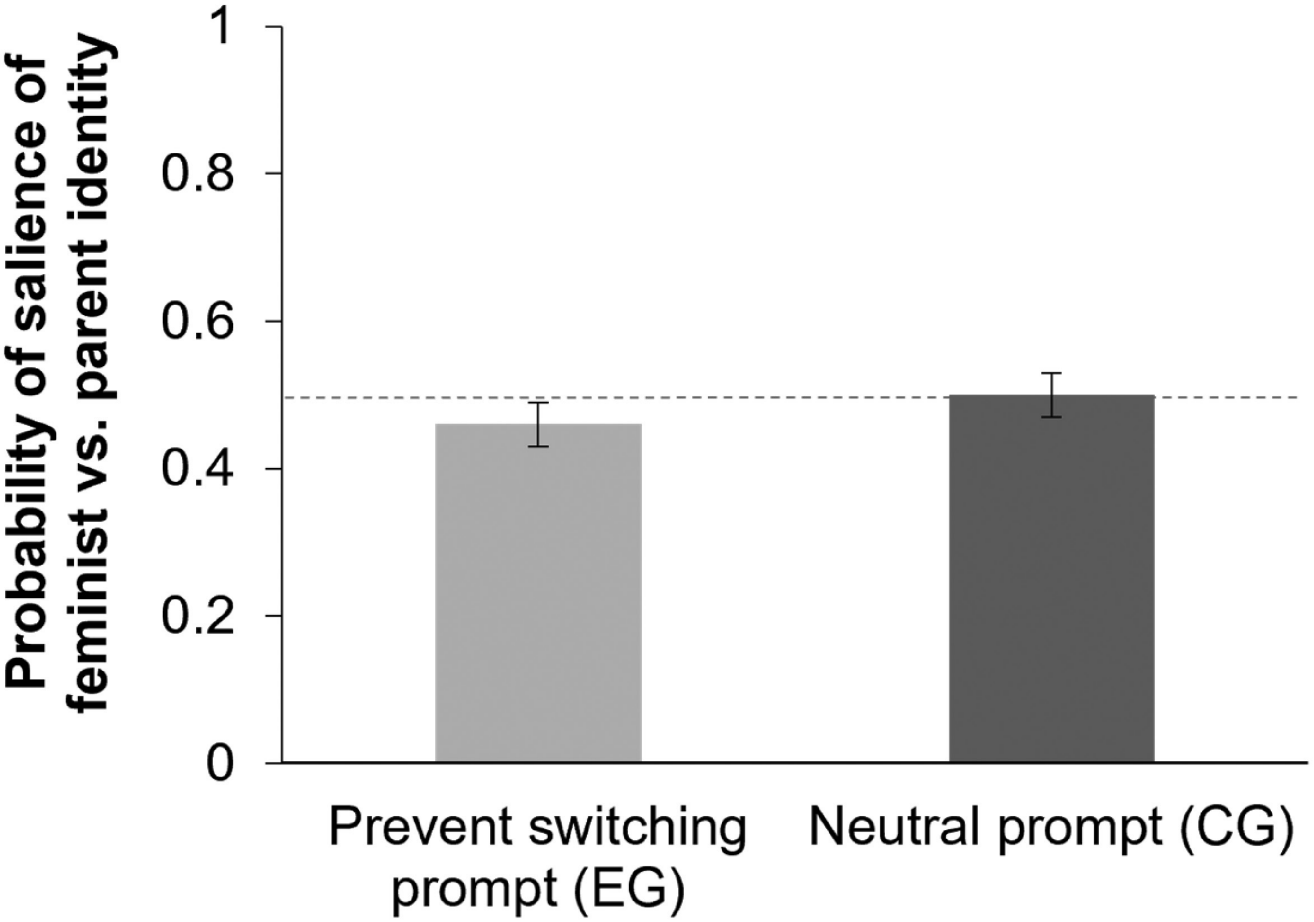
Salience of feminist versus parent identity by condition. *Note*: A score of 1 indicates that it is extremely likely that a parent identity rather than a feminist identity is salient; a score of 0 indicates that it is extremely likely that a feminist rather than parent identity is salient; and 0.5 indicates that no clear distinction between salience of the feminist or parent identity can be made (indicated by the reference line).

An independent samples *t*‐test, which examined whether participants in the experimental group revealed in their writing style greater parent‐relative‐to‐feminist identity salience than those in the control group, revealed no significant difference between the two groups, *t*(183) = 0.89, *p* = .377, *d* = 0.13, see Figure 2. Therefore, the hypothesis that participants are able to prevent social identity switches cannot be supported based on the implicit social identity measure.

##### H2 – explicit measure

**FIGURE 3:**
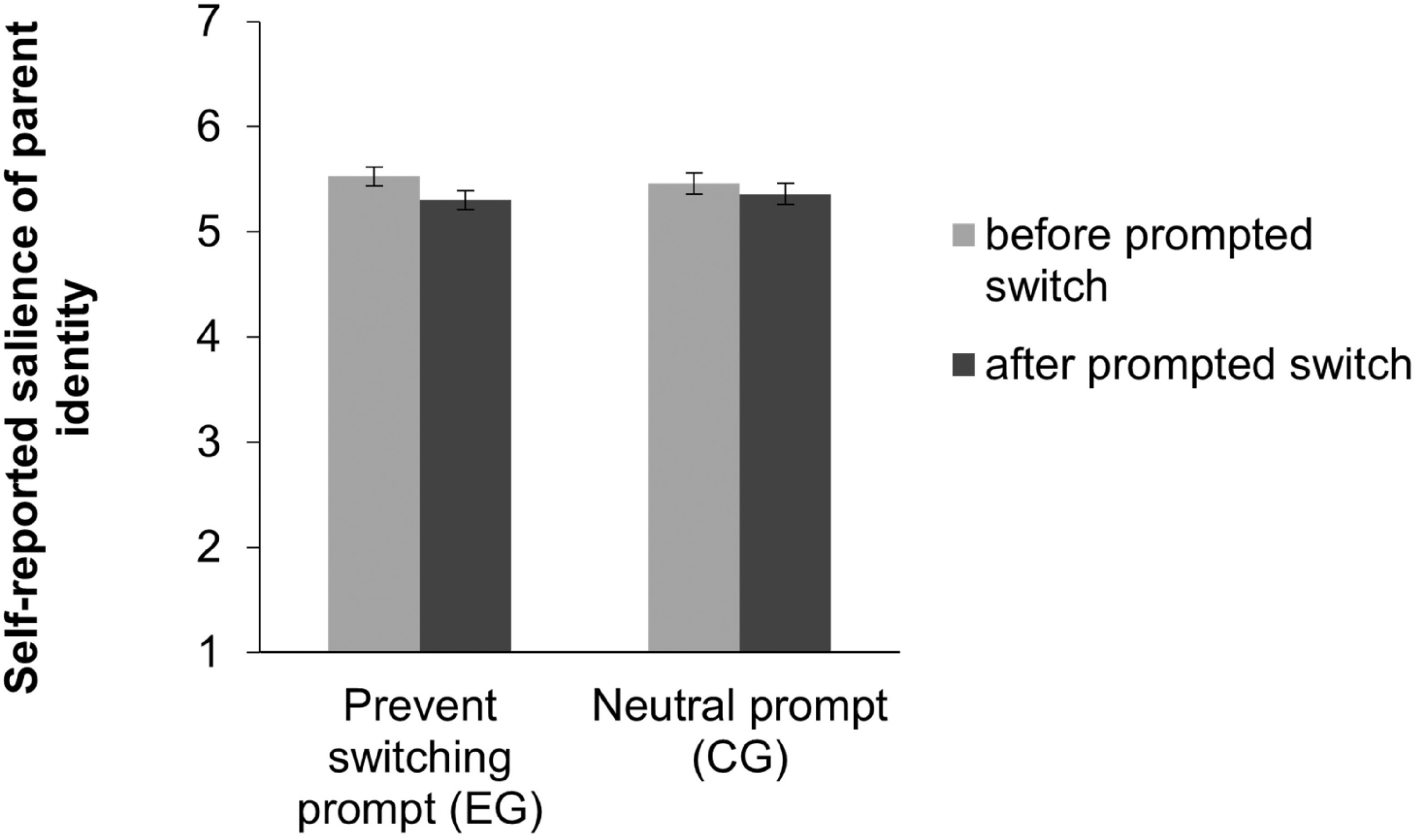
Self‐reported salience of parent identity depending on condition and time.

We tested whether participants in the experimental group showed less reduction in self‐reported salience of the parent identity than participants in the control group at T2 relative to T1. We conducted a 2 × 2 mixed‐measures ANOVA with condition (group) and time (T1 vs. T2).^5^ As shown in Figure 3, we found the expected main effect of time, *F*(1, 183) = 8.49, *p* = .004, $\eta_{p}^{2}$ = .044 with an overall reduction in self‐reported salience of the parent identity from before (*M* = 5.49, *SE* = 0.06) to after (*M* = 5.33, *SE* = 0.07) the prompted switch, showing that a switch was prompted successfully; there was no significant main effect of condition, *F*(1, 183) = 0.003, *p* = .960, $\eta_{p}^{2}$ < .001. There was no significant interaction between condition and time, *F*(1, 183) = 1.33, *p* = .251, $\eta_{p}^{2}$ = .007 and, therefore, no support for the hypothesis that participants prevented the identity switch.

#### Follow‐up analysis

One strategy that participants might have used to remain in their parent identity is to write about the required topic from the perspective of a parent. We used LIWC (Pennebaker et al., 2007) to extract the number of family‐related words used in the main writing task. Participants instructed to stay in their parent identity (*M* = 1.00, *SE* = 0.15) used significantly more family‐related words than participants in the control condition, *M* = 0.31, *SE* = 0.08; *t*(183) = −4.02, *p* < .001, *d* = 0.59. Pearson correlations were conducted using Bonferroni‐adjusted alpha levels of .025 per test (.05/2) to correct for the inflation of familywise error to test for a positive relationship between the number of family‐related words and the change in self‐reported salience from T1 to T2. Neither the change in the salience of the parent identity from T1 to T2 in the control group, implicit: *r*(92) = −.05, *p* = .623, self‐reported: *r*(92) = −.15, *p* = .162 nor in the experimental group, implicit: *r*(93) = −.09, *p* = .375, self‐reported: *r*(93) = .01, *p* = .913 approached statistical significance, suggesting that the use of family‐related words did not increase or maintain the salience of the parent identity.

### Discussion

The aim of this study was to establish whether participants can prevent social identity switches. The linguistic style of participants instructed to keep thinking of themselves as parents neither did not show a higher probability of having a parent identity salient relative to the salience of their feminist identity nor did these participants show a smaller reduction in the self‐reported salience of the parent identity than the control group. The significant main effect of time in the explicit measure confirms that the writing topic was effective in prompting participants to switch away from their parent identity. These findings suggest that participants were not able to prevent a social identity switch elicited by the topic of the writing task, despite being instructed to do so. Follow‐up analyses showed that participants in the experimental group used more family‐related words than participants in the control group. However, this strategy was not sufficient to prevent a switch. According to the results from the linguistic style analysis, the fact that a person uses more parent‐ or family‐related words does not necessarily mean that this person is thinking of themselves as a parent.

It can be argued that the participants' motivation to prevent switching identities was relatively low. Participants in the experimental group were only presented with a short instruction to try to stay in their parent identity prior to the prompted switch. This instruction might have been overlooked or given low priority by the participants. It is therefore conceivable that participants may be able to control social identity switches if they are subject to a stronger motivational manipulation such as a monetary incentive.

Alternatively, it is possible that the task of writing on a topic about the objectification of women did not induce a strong enough switch from the parent to the feminist identity in either of the two groups of participants, which may have obscured attempts to prevent the switch in the experimental group. Although the self‐report measure suggested that the writing task did prompt an increase in the salience of the feminist identity, the implicit (linguistic) measure is somewhat equivocal in this regard – the probability values for both groups in Figure 2 are very close to 0.5 (indeterminate), especially in the control group where one would expect a clearer switch to the feminist identity elicited by the writing task. A baseline implicit measure (before writing about objectification of women) would have been useful in this regard because it would have allowed us to determine the change in the salience of the feminist identity elicited by the writing manipulation.

To address the above issues, we conducted the second study to: (1) provide a material incentive/motivation to prevent the social identity switch, (2) acquire the implicit (linguistic) measure before and after the task of writing about objectification of women and (3) increase the sample size to test for a smaller‐than‐medium effect of the attempt to prevent a social identity switch.

## STUDY 2 – PREVENTING A SOCIAL IDENTITY SWITCH WHEN INCENTIVIZED TO DO SO

Monetary incentives, in the form of performance‐based bonuses, have been successfully used to augment the effort and performance, and increase the allocation of cognitive control in experimental studies (Capa et al., 2013; Capa & Bouquet, 2018), including task‐switching studies (Lavric et al., 2008; Longman et al., 2016, 2017, 2021; Nieuwenhuis & Monsell, 2002). Capa et al. (2013) reported a relatively long‐lasting effect of the incentives on participants' performance, finding that the rewards also increased participants' preparatory effort. Hence, in Study 2, participants were incentivized to stay in a social identity (the parent identity). A further important change in Study 2 is the addition of a linguistic measure at baseline (before the prompted switch). This allows us to compare the implicit salience measures at two time points to determine the change in salience from before to after the prompted switch.

### Method

#### Participants

Based on sample size calculations (see Appendix D), the target sample for recruitment was *N* = 382 (minimum target of 200; 100 participants per group).^6^ Participants were recruited and reimbursed through Prolific. The same inclusion criteria as in Study 1 were used and, additionally, we only invited participants who had not taken part in Study 1. Participants were paid at least £1.50 for participation. Participants in the experimental group could earn an extra £1 performance‐related bonus payment.

As in Study 1, we excluded participants who wrote less than 25 words in the main writing task or did not complete the second salience self‐report measure. The final sample size was *N* = 375 (control group: *n* = 188; experimental group: *n* = 187). Participants were on average 43.12 years old (*SD* = 12.27, min = 20, max = 77); 66.1% identified as female (male = 33.3%; other = 0.6%); the majority indicated being either UK (40.8%) or US (32.3%) nationals (7.2% South African, and 19.7% other nationalities each under 5% of participants).^7^ The study was approved by the departmental ethics committee at the University of Exeter.

#### Design

The study has a mixed design, with condition (prompt to prevent switch vs. neutral prompt) as a between‐participants factor and the time of the measurement (before vs. after the switch) as a within‐participant factor. The dependent variables are parent/feminist identity salience measured with the ASIA classifier (implicit measure) and self‐reported identity salience (explicit measure).

#### Materials and procedure

The study's procedure was modelled on the procedure of Study 1. Participants were randomly allocated to the experimental group or the control group. The social identity switch was prompted by asking participants to write about the same topic linked to their feminist social identity as in Study 1.

##### Implicit salience measure

As in Study 1, participants completed a three things manipulation (Haslam et al., 1999) to activate their parent identity. However, instead of being asked to provide only keywords to the questions, participants had to write a short text (4–5 sentences) as part of the three things manipulation. In addition to analysing the identity salience during the main writing task (used to prompt the identity switch), the short text that participants wrote as part of the three things manipulation (Haslam et al., 1999) allowed us to analyse the linguistic style prior to prompting the identity switch. The identity salience during both writing tasks was assessed with the parent–feminist ASIA (Koschate et al., 2021), as explained in Study 1. To adapt the classifiers, we used domain adaptation (Fernando et al., 2013). It was based (for both reported studies) on the experimental data from the three things writing task in Study 2, which provided a baseline measure of linguistic style (for more information, see Appendix B).

##### Explicit salience measure

To assess the change in self‐reported salience, participants were asked to rate the statement ‘I am thinking of myself as a parent right now’ (based on Verkuyten & Hagendoorn, 1998) on a 7‐point Likert scale from 1 (strongly disagree) to 7 (strongly agree) twice – immediately after the three things manipulation and after the main writing task.

##### Switch prevention manipulation

The prompts in both groups were rephrased to include an additional sentence to improve the clarity of the instruction in the experimental group: ‘We all wear different “hats” in our everyday lives, and sometimes specific situations can prompt us to think of ourselves in a specific role’. A further departure from Study 1 was that participants in the experimental group received an incentive to stay in their parent identity outlined in the following message: ‘Important: We will use statistical analysis to detect whether people managed to keep thinking of themselves as a parent. The 50% of people that best managed to do so will receive an additional reward payment of £1 on Prolific’. The incentive mentioned ‘statistical analysis’ rather than the linguistic style analysis to prevent participants from trying to alter their linguistic style. Participants received the bonus payment on Prolific (allocated through their anonymous Prolific ID) after data collection for the study was completed and the relevant data were analysed to determine which participants best managed to remain in their parent identity.

##### Demographics and identification questionnaire

The demographics and identification questionnaire participants completed at the end of the study (see Supporting Information: S2) was based on a shortened version of the questionnaire used in Study 1 (see Appendix E for a summary of measures and sample descriptives).^8^

### Results

#### Preliminary analysis

##### Randomization checks

Chi‐square tests of independence showed that the two groups did not differ in gender distribution, *χ* ^2^(1, *N* = 373) = 0.26, *p* = .609; male vs. female and with regard to children currently living at home or not, *χ* ^2^(1, *N* = 374) = 0.02, *p* = .886. There was no significant difference between the two groups in: participant's age, *t*(373) = 0.38, *p* = .706, *d* = 0.04; age of the youngest child, *t*(372) = −0.18, *p* = .858, *d* = 0.02; and word count in the main writing task, *t*(373) = −1.02, *p* = .307, *d* = 0.11. Most importantly, the two groups were not significantly different in their linguistic style in the first text, prior to the prompt; *t*(353) = 0.89, *p* = .375, *d* = 0.09 and in their first self‐reported salience score of the parent identity, *t*(373) = −0.37, *p* = .710, *d* = 0.04.

#### Hypothesis testing

##### H1 – implicit measure

**FIGURE 4:**
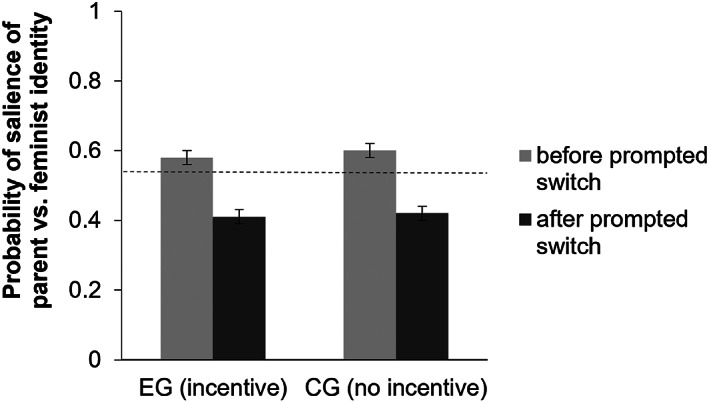
Salience of feminist versus Parent identity depending on condition and time. *Note*: A score of 1 indicates that it is extremely likely that the parent rather than feminist identity is salient; 0 indicates that it is extremely likely that a feminist rather than parent identity is salient; and 0.5 indicates that no clear distinction between salience of the feminist or parent identity can be made (indicated by the reference line).

The exclusion of participants who wrote less than 25 words in the three things writing task resulted in *N* = 355 for this specific analysis. We conducted a 2 (condition) × 2 (time) mixed‐measures ANOVA (Figure 4) to test whether participants in the experimental group showed less change in salience from a parent to a feminist identity between *T*1 (after the three things task) and *T*2 (writing the text on the feminism‐related topic) than participants in the control condition (H1). The main effect of condition was not significant, *F*(1, 353) = 0.44, *p* = .506, $\eta_{p}^{2}$ = .001. We found a significant main effect of time, *F*(1, 353) = 70.83, *p* < .001, $\eta_{p}^{2}$ = .167 which reflected a higher probability of the parent identity being salient rather than the feminist identity before (*M* = 0.59, *SE* = 0.02) compared to after (*M* = 0.41, *SE* = 0.02) the prompted switch. As shown in Table 1, there is a significant difference in implicit identity salience between *T*1 and *T*2 in both the experimental group and the control group, indicating that both groups switched from a parent to a feminist identity. Crucially, we found no significant interaction between condition and time, *F*(1, 353) = 0.28, *p* = .595, $\eta_{p}^{2}$ = .001. Therefore, based on the ASIA measure, we found that a switch was successfully triggered between *T*1 and *T*2, but we found no support for H1.

**TABLE 1:** Within‐group follow‐up analyses for explicit and implicit salience measure at T1 and T2.

Measure	Group	*T*1 *M*	*T*2 *M*	*M*_Diff_	*SE*	*t*	*df*	*p*	*d*
Explicit (ASIA)	Switch prevention incentive	0.58	0.41	0.17	0.03	5.66	178	<.001	0.42
Explicit (ASIA)	No prevention prompt/incentive	0.60	0.42	0.19	0.03	6.23	175	<.001	0.47
Implicit (Self‐report)	Switch prevention incentive	6.68	6.49	0.19	0.07	2.70	186	.008	0.20
Implicit (Self‐report)	No switch prompt/incentive	6.65	5.60	1.05	0.13	8.15	187	<.001	0.59

##### H2 – explicit measure

**FIGURE 5:**
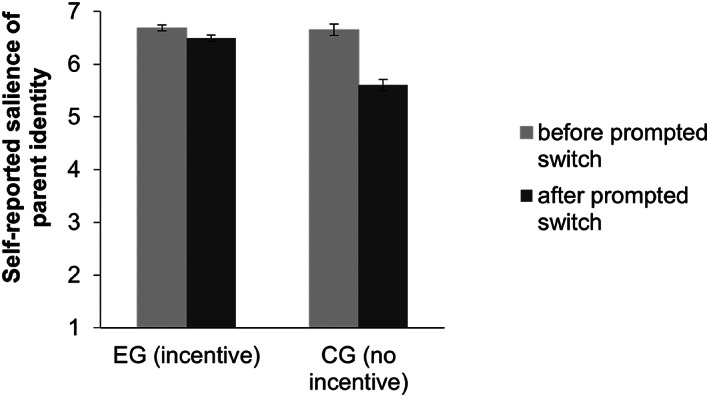
Self‐reported salience of parent identity depending on condition and time.

A 2 (condition) × 2 (time) mixed‐measures ANOVA tested whether the experimental group showed a smaller decrease in self‐reported salience of the parent identity than the control group. There was a significant interaction between condition and time, *F*(1, 373) = 33.88, *p* < .001, $\eta_{p}^{2}$ = .083. As shown in Figure 5, participants in the experimental group showed a weaker (although still significant; see Table 1) decrease in self‐reported salience of the parent identity than participants in the control group. We found a significant main effect of condition, *F*(1, 373) = 22.95, *p* < .001, $\eta_{p}^{2}$ = .058 with overall higher salience scores for the parent identity reported in the experimental (*M* = 6.59, *SE* = 0.07) than in the control group (*M* = 6.13, *SE* = 0.07). The main effect of time was also significant, *F*(1, 373) = 70.98, *p* < .001, $\eta_{p}^{2}$ = .160, with higher parent identity salience scores before (*M* = 6.67, *SE* = 0.04) compared to after (*M* = 6.05, *SE* = 0.08) the prompted switch. The effect of time was significant in both the experimental and control group (Table 1).

To account for the differences in the sample used for this analysis as compared to the sample used for the analysis of the linguistic style (implicit) measure, we re‐ran the analyses of the explicit measure after excluding participants who wrote less than 25 words in the three things writing task. Excluding those participants did not change the pattern of results (see Table 2).

**TABLE 2:** 2 (Condition) × 2 (Time) ANOVA results for reduced sample size *n* = 355.

Effect	*F*	*df*	*p*	$\eta_{p}^{2}$
Condition	25.30	1, 353	<.001	.067
Time	72.07	1, 353	<.001	.170
Condition × Time	38.62	1, 353	<.001	.099

#### Follow‐up analysis

As in Study 1, we analysed the number of family‐related words in the main writing task. Participants in the experimental group (*M* = 1.32, *SE* = 0.13) used significantly more family‐related words than participants in the control group, *M* = 0.24, *SE* = 0.05; *t*(373) = −7.68, *p* < .001, *d* = 0.79. Pearson correlations with Bonferroni‐adjusted alpha levels of .025 per test (.05/2) found no significant relationship between the number of family words and the change in the salience of the parent identity from T1 to T2 in the control group, implicit: *r*(174) = .07, *p* = .343, explicit: *r*(188) = .07, *p* = .347 or the experimental group, implicit: *r*(178) = .11, *p* = .141, explicit: *r*(187) = .16, *p* = .027.

### Discussion

The results of Study 1 suggested that participants could not prevent a switch of social identity elicited by the topic about which they had to write. However, it is conceivable that (some) participants might not have paid sufficient attention to the instruction to remain in their parent identity or were not sufficiently motivated to prevent the switch, or the text writing task was not sufficiently effective in eliciting the switch. Therefore, the main aims of Study 2 were to test whether participants may be able to do it if offered a performance‐related monetary bonus and acquire a baseline implicit measure of linguistic style to enable a better estimate of the effectiveness of text writing in eliciting the switch.

The comparison of the implicit salience measure before and after the switch task validates the design of this study (and, by extension, that of Study 1) by showing that the writing topic in the main task indeed prompted a switch from the parent to the feminist identity and that the linguistic classifier could distinguish between the salience of the two identities before versus after the switch. Despite the incentive, the analysis of linguistic style found no evidence that participants could prevent the switch from the parent to the feminist identity. Yet, the self‐report measure indicated that participants instructed and incentivized to remain in their parent identity reported a smaller reduction in the salience of the parent identity than participants in the control group. As in Study 1, participants in the experimental condition used more family‐related words but this did not correlate with the salience measures.

We interpret these findings to mean that participants in the experimental group were aware of the task requirement and felt that they were able to keep their parent identity salient, as shown by the explicit measure. However, the implicit salience measure revealed no significant difference between the two groups, despite detecting robustly the identity switch in both groups of participants. Therefore, the participants' insight into their ability to prevent a social identity switch seems to stand in contrast to the actual ability to do so. We consider potential reasons for this discrepancy in the General Discussion.

## GENERAL DISCUSSION

Theory and previous research (Zinn et al., 2022) suggest that social identity switches are highly effective and relatively rapid. Are switches so effective because they are strongly environmentally driven, or because participants' intentional control of identity salience is highly effective? The present studies asked if participants could prevent switching social identities when instructed to do so. In addition to the explicit instruction, participants also received a monetary incentive to prevent the switch in Study 2. Social identity salience was assessed with a classifier‐based analysis of participants' linguistic style and an explicit self‐report measure.

The results of Study 1 showed that participants could not avoid a switch to a social identity prompted by writing a text. For the implicit salience measure, this limitation of intentional (top‐down) control was also found in Study 2 even when participants were specifically incentivized to stay in the same identity. Furthermore, since previous research has shown that ASIA can distinguish between the parent and feminist identity even when the writing topic is linked to the feminist identity (Koschate et al., 2021), the current findings can be clearly attributed to limited control over social identity switches.

As in Study 1, both groups also showed a significant decrease of the self‐reported salience of the parent identity. However, participants in the experimental group in Study 2 indicated that they thought they had prevented the switch, at least to some extent. These findings show that even when people report that they did so, the actual intentional control over social identity switches may be limited. We note that self‐report measures in the presented study could have been influenced by demand characteristics and that participants might have limited insight into which identity is salient at a given time (Koschate et al., 2021; Nisbett & Wilson, 1977). In Study 2, participants in the experimental group were informed that they would receive a bonus payment for remaining in their parent identity. This could have led to (or increased) the demand characteristic of reporting a persistently salient parent identity. Furthermore, due to the demand to stay in their parent identity, those participants might have switched back to this identity after the writing task. This would explain why only the implicit measure – which assesses salience throughout the writing task rather than once it is completed – shows the limited ability to prevent switches. These differences between explicit and implicit salience measures in Study 2 highlight the importance of including measures of salience (such as ASIA) which are independent of the participants' introspections and their perceptions of researchers' expectations and measure salience during the task itself.

### Theoretical and practical implications

Testing the extent to which social identity switches can be prevented or limited has important theoretical implications. Some authors have implied that people have intentional control over social identity switches and that this, in turn, helps them to adapt to their surroundings (Hugenberg & Bodenhausen, 2004; van Bavel & Packer, 2021). However, the present evidence suggests that people may not be able to prevent social identity switches when the environmental trigger (here, the topic of a writing task) elicits a switch. We are also not aware of other evidence to support the notion that people have intentional control over their salient identity.

Our results seem to be at odds with findings from the task‐set control literature, reviewed earlier, which indicate that even processes commonly thought as automatic, such as word identification, are at least to an extent, subject to intentional task‐set control (Elchlepp et al., 2015, 2017). We note, however, that task‐switching studies have also identified processes which seem to be independent of the top‐down task‐set. In particular, Elchlepp et al. (2021) provided EEG evidence that participants discriminated facial emotional expressions to a similar degree when it was task relevant and when it was irrelevant (and even detrimental) for the performance of the task (identifying a letter superimposed on the face).

Our studies show that rapid and effective social identity switches (Zinn et al., 2022) might come with a ‘downside’ in the form of limited top‐down control over switches. This limit in intentional control may have important implications for how people manage the different identities in their lives. Specifically, our finding indicates that an identity switch might not be preventable when it is triggered by environmental demands. This highlights the strength of contextual factors in determining which identity becomes salient at a given moment (Oakes, 1987).

### Limitations and future research

Importantly, the current studies focus on a relatively strong prompt to switch identities. First, participants had to attend to the switching prompt because it was integrated into the writing task. In other words, participants could not block out the feminist identity prompt using selective attention at the perceptual stage. Arguably, many triggers in someone's surroundings may potentially be ‘filtered out’ via selective attention mechanisms to avoid an identity switch. For instance, while writing a work‐related email a parent might hear their children play in the background but successfully tune‐out their auditory perception to prevent switching to the parent identity. Our research takes an important first step in testing whether people can control identity switches when attending to (and processing of) the switching prompt is mandatory (unavoidable). Future research is needed to establish whether attentional filtering of prompts to switch is a successful strategy in preventing identity switches. This could be investigated, for instance, by using a neutral text while cueing a specific identity through additional visual or auditory feedback – a scenario similar to the discussed example of writing a work email while hearing one's children play in the background.

Second, participants might have found it especially difficult to remain in their parent identity as the context (writing about a feminist topic) included no stimuli or context related to their parent identity. Participants might be able to prevent the switch if the environment, or current activity, includes cues related to the previous identity. In the current studies, it could be argued that for the writing task, it was potentially more optimal for participants to make the feminist identity salient – in order to complete the writing task. This differs from the example, mentioned earlier, of a doctor treating a child. In the example, the optimal identity would likely be the professional identity, which could make it easier for the doctor to prevent switching away from it. Taken together, our findings suggest that people have limited control over switches elicited by prompts whose processing cannot be avoided or is strongly reduced. Going forward, it will be important to investigate whether any effect of participants' top‐down control processes can be found for other switching prompts.

Referring back to SCT (Turner et al., 1987), an important theoretical and practical avenue for future research will be to account for different factors known to drive salience: perceiver readiness; comparative and normative fit (Oakes, 1987). Perceiver readiness encompasses intrapersonal factors that can lead to an identity switch such as people's past experiences and current goals, needs and motives (Turner et al., 1994). While our studies suggest that people have limited control, it might be possible to control switches if there is a stronger motive or need to stay in a specific identity. Further, individual differences might arise from people differing in their past experience of activating a specific identity.

In contrast to intrapersonal factors captured by perceiver readiness, comparative and normative fit relate to the social context. Comparative fit is informed by the meta‐contrast principle (Turner et al., 1987): stimuli are more likely to be perceived as a group if they share more characteristics with each other than with stimuli outside the group. Normative fit focuses on the category content – in order to be classified as a group, shared characteristics have to meet normative beliefs held about the relevant social identity (Turner et al., 1994). The current studies did not manipulate these factors separately. Therefore, it will be important to test how exactly these factors interact in influencing whether an identity switch can be controlled or not. Future studies could investigate whether participants can exert top‐down control to initiate a switch towards an identity even though comparative and normative fit are manipulated to be low.

## CONCLUSION

To conclude, our findings illustrate that people may have limited top‐down control over social identity switches. The implicit salience measure showed that participants could not successfully prevent switching identities even when incentivized to do so and even when participants reported to have done so (in Study 2). Our paradigm and results open up avenues for future research on top‐down control over social identity switches.

## AUTHOR CONTRIBUTIONS

**Anna K. Zinn:** Conceptualization; data curation; formal analysis; funding acquisition; investigation; methodology; project administration; software; supervision; validation; visualization; writing – original draft; writing – review and editing. **Miriam Koschate:** Conceptualization; funding acquisition; methodology; supervision; validation; visualization; writing – review and editing. **Elahe Naserianhanzaei:** Data curation; formal analysis; methodology; software. **Aureliu Lavric:** Conceptualization; funding acquisition; methodology; supervision; validation; visualization; writing – review and editing.

## CONFLICT OF INTEREST STATEMENT

The authors declare no conflict of interest.

### OPEN RESEARCH BADGES

This article has earned Open Data, Open Materials and Preregistered Research Design badges. Data, materials and the preregistered design and analysis plan are available under the below links: Data and materials: osf.io/x34a6/;

Pre‐registration Study 1: osf.io/asjv8

Pre‐registration Study 2: osf.io/tavd8.

## Supporting information


Data S1.

## Data Availability

The data that support the findings of both studies is openly available on OSF (project doi: 10.17605/OSF.IO/X34A6). OSF link: osf.io/x34a6/

## Footnotes

^1^
The hypotheses are in line with the hypotheses pre‐registered on OSF. H2 has been slightly rephrased to increase clarity.

^2^
OSF links to pre‐registrations, data and materials are listed at the end of the manuscript.

^3^
For a summary of additional sample descriptives see Appendix C.

^4^
These measures are presented in the Appendix only as they were not part of the main hypothesis testing.

^5^
The OSF pre‐registrations proposed independent sample *t*‐tests to test all hypotheses. We decided to run a repeated measures ANOVA to gain a better understanding of the effect of time (for H2 in Study 1 and H1 and H2 in Study 2).

^6^
OSF links to the pre‐registrations, data and materials are listed at the end of the manuscript.

^7^
For a full summary of sample descriptives, see Appendix D.

^8^
These measures are presented in the Appendix only as they were not part of the main hypothesis testing.

## APPENDIX A

**Study 1: Sample size calculations**

The required sample size was calculated with the program G*Power (Faul et al., 2007). The aim was to recruit at least 176 participants (a minimum of 88 participants per group) to obtain .95 power at the standard .05 alpha error probability and with *d* = 0.50 (for the independent samples *t*‐test for the implicit salience measure). For the explicit salience measure, we ran a 2 (condition: prompt to prevent switch vs. neutral prompt) × 2 (time: before vs. after switch) mixed‐measures ANOVA. However, as we were interested in the interaction effect, the required sample size would be equivalent to the calculations for an independent samples *t*‐test on the difference between the time points.

## APPENDIX B

**Domain adaptation**

To adapt the classifiers in both Study 1 and Study 2, we used domain adaptation (DA; Fernando et al., 2013). As outlined by Koschate et al. (2021), it is important to account for differences in distribution between the initial training dataset from an online forum and test datasets obtained as part of an experiment. These differences may stem from changes in task requirements (e.g. our experimental instruction to write 3–5 sentences). This difference in distributions can lead to an overall shift in scores for experimental datasets, which can be corrected for by using DA. DA takes into account both the initial training dataset and a dataset of the new domain (e.g. experimental data). In Studies 1 and 2, we started with the classifier trained on forum posts on Mumsnet (Koschate et al., 2021) and adapted based on participants' responses to the three things writing task in Study 2. The three things writing task was expected to lead to participants adopting either their feminist or parent identity and can therefore function as a baseline measure (‘ground truth’) for the salient identities. The adapted model was then used to extract the probability for the three things writing task as well as the main writing tasks in both Study 1 and Study 2.

## APPENDIX C

**Study 1: Measures and sample description**

The full questionnaire included: demographic questions (about participants' age, language, gender and nationality); questions about participants' parental identity (the age of their youngest child and whether they currently have children living with them); one item assessing conflict between the parent and feminist identity (based on Benet‐Martínez & Haritatos, 2005); four social identification items by Doosje et al. (1995) and one item assessing social identification based on Haslam et al. (1999) which were combined to form a 5‐item scale of social identification (for parent identity: *α* = .76; for feminist identity: *α* = .88).

Furthermore, participants in the experimental group were also asked to indicate how difficult they found it to keep thinking of themselves as a parent (‘How difficult did you find it to keep thinking of yourself as a parent during the writing task?’; 1 = very difficult to 4 = very easy; the data were reverse scored so that higher scores reflected a higher difficulty) and to estimate the effort it took to prevent switching – the latter was done using the item ‘I tried very hard to think about myself as a parent when completing the writing task’ (1 = strongly agree to 4 = strongly disagree; for analysis, the data were reverse scored so that higher scores reflected a higher effort). Identity prototypicality measures were included for both identities (Fielding & Hogg, 1997), as well as items that asked participants whether they perceived the topic to be typical for feminists or parents and whether the topic made them think of themselves as a parent or feminist. Participants were also asked whether they managed to think about themselves as a parent at the start of the study and after the writing task. These measures were included for potential exploratory analyses but were not part of the main hypotheses testing.

All participants indicated English as their mother tongue; 8.1% were bi‐ or multilingual, indicating English as one of their languages. The mean age of participants' youngest child was 9.66 years (*SD* = 9.43, min = 0, max = 44); 86.5% of participants reported that they had children living at home with them. Overall, participants self‐reported identifying significantly more strongly, *t*(183) = 2.12, *p* = .036, *d* = 0.16 with their parent identity (*M* = 5.93, *SD* = 0.87) than their feminist identity (*M* = 5.73, *SD* = 0.97). Participants reported low conflict scores between the feminist and parent identity (*M* = 2.12, *SD* = 1.62).

## APPENDIX D

**Study 2: Sample size calculations**

We based our sample size calculations on Brysbaert (2019). Our previous study indicates that the effect of control over social identity switches might be small (*d* = 0.20). However, the current study altered the paradigm by adding an incentive to prevent the switch. Therefore, we expected a small‐to‐medium effect. Due to the added baseline measure for the implicit salience, we ran 2 (condition) × 2 (time) mixed‐measures ANOVAs for both the implicit and explicit salience measures, testing for the interaction effect (sample size calculations would be equivalent to the calculations for an independent samples *t*‐test on the difference between the times). At least 100 participants would be required per group for a *t*‐test between groups with a power of .80 and a small‐to‐medium effect size *d* = 0.40 (Brysbaert, 2019). Based on these calculations, we aimed to test a minimum of 200 participants (100 per group) but with the aim to recruit up to 382 participants, if possible (based on G*Power calculations; Faul et al., 2007), this would allow us to test for a smaller effect size of *d* = 0.30 with an increased power of .90.

## APPENDIX E

**Study 2: Measures and sample description**

The following measures from Study 1 were also included in Study 2: all demographic items, self‐reported effort (experimental group only) and difficulty (experimental group only) to remain in parent identity, identity conflict, social identification.

All items in the questionnaire were assessed on 7‐point Likert scales. The five‐item identification scale was reliable for both identities (for parent identity: *α* = .79; for feminist identity: *α* = .95). An additional effort item was included for the control group, asking participants to respond on a 7‐point Likert scale (1 = strongly disagree; 7 = strongly agree) to the statement ‘I tried very hard to think of strong arguments for the second writing task’.

Regarding the language profile, all participants had English as their mother tongue; 6.9% of the participants were bi‐ or multilingual and listed English as one of the languages. The mean age of participants' youngest child was 11.89 years (*SD* = 10.86, min = 0, max = 53). The majority of the participants (84.5%) reported that they currently have a child/children living at home with them. Participants reported a significantly stronger, *t*(372) = 12.26, *p* < .001, *d* = 0.64 identification with their parent identity (*M* = 6.21, *SD* = 0.83) than their feminist identity (*M* = 5.19, *SD* = 1.50). Furthermore, participants reported low conflict scores for the two identities (*M* = 2.39, *SD* = 1.66).
