# Supplementary material for: Can we prevent social identity switches? An experimental–computational investigation
Source: Br J Soc Psychol. 2023 Apr 11;62(3):1547–65. doi: 10.1111/bjso.12647 (PMC10947443; doi:10.1111/bjso.12647)
Supplement: Supplementary file 1 — Data S1. [file BJSO-62-1547-s001.docx]

**Supplementary Materials for**

**Can we prevent social identity switches? An experimental-computational investigation**

**This file includes:**

- S1: Study 1 Demographics and Identification questionnaire
- S2: Study 2 Demographics and Identification questionnaire

## S1: Study 1: Demographics and identification questionnaire

**Please answer a few questions about the tasks:**

 The questions below refer to the writing task:

|  | Parent(s) | |  |  | | Feminist(s) | |
| --- | --- | --- | --- | --- | --- | --- | --- |
| The topic ‘Objectification of Women’ is typical for: |  |  |  |  |  | |  |
| The topic made me think of myself as a: |  |  |  |  |  | |  |

I managed to think of myself as a parent at the start of the study

o Definitely

o Probably

o Probably not

o Definitely not

Did you still think of yourself as a parent during the writing task? [* CG only]

o Definitely

o Probably

o Probably not

o Definitely not

I tried very hard to think about myself as a parent when completing the writing task: [*EG only]

o Strongly Agree

o Agree

o Disagree

o Strongly Disagree

How difficult did you find it to keep thinking of yourself as a parent during the writing task? [*EG only]

o Very Difficult

o Difficult

o Easy

o Very Easy

**Please indicate how strongly you agree or disagree with the following statements:**

|  | Do not agree at all | |  |  |  | Agree Completely | |
| --- | --- | --- | --- | --- | --- | --- | --- |
| I see myself as a parent |  |  |  |  |  |  |  |
| I am pleased to be a parent |  |  |  |  |  |  |  |
| I feel strong ties with other parents: |  |  |  |  |  |  |  |
| I identify with other parents |  |  |  |  |  |  |  |
| Being a parent is an important reflection of who I am |  |  |  |  |  |  |  |

|  | Do not agree at all | |  |  |  | Agree Completely | |
| --- | --- | --- | --- | --- | --- | --- | --- |
| I see myself as a feminist |  |  |  |  |  |  |  |
| I am pleased to be a feminist |  |  |  |  |  |  |  |
| I feel strong ties with other feminists |  |  |  |  |  |  |  |
| I identify with other feminists |  |  |  |  |  |  |  |
| Being a feminist is an important reflection of who I am |  |  |  |  |  |  |  |

|  | Do not agree at all | |  |  |  | Agree Completely | |
| --- | --- | --- | --- | --- | --- | --- | --- |
| I feel conflicted between being a parent and being a feminist |  |  |  |  |  |  |  |

**Please now consider the behaviours, qualities, goals, and norms that characterise [parents/ feminists] and differentiates them from other groups.**

Please sum up the qualities that best described the character, style, and spirit of [parents/femnists] (i.e., the prototype) in no more than five lines.

___________________________________________________________________________

___________________________________________________________________________

___________________________________________________________________________

___________________________________________________________________________

___________________________________________________________________________

|  | How easy did you find it to generate this description? | | | | | | | | |  |
| --- | --- | --- | --- | --- | --- | --- | --- | --- | --- | --- |
| not very easy |  |  |  |  |  |  |  |  |  | very easy |

|  | How confident are you that this description is accurate? | | | | | | | | |  |
| --- | --- | --- | --- | --- | --- | --- | --- | --- | --- | --- |
| not very confident |  |  |  |  |  |  |  |  |  | very confident |

|  | How well do you think you fit this description? | | | | | | | | |  |
| --- | --- | --- | --- | --- | --- | --- | --- | --- | --- | --- |
| not at all |  |  |  |  |  |  |  |  |  | very much |

**What percentage of the group do you think would agree with this description?**

|  | 0 | 10 | 20 | 30 | 40 | 50 | 60 | 70 | 80 | 90 | 100 |
| --- | --- | --- | --- | --- | --- | --- | --- | --- | --- | --- | --- |

| Percentage | 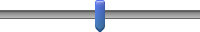 |
| --- | --- |

**Almost done, please answer a few short questions about yourself:**

How old are you? ________

What is your first language?

- English is my first language
- Other (please specify) ____
- I am bi-/multilingual (please specify the 2 languages you are most proficient in) _______

Which term best describes your gender?

- Male
- Female
- Non-binary
- Other (please specify) _____________

Please indicate your nationality

- Australian
- British
- Other (please specify) _______________

How old is your youngest child? ________________

Do you currently have children living with you?

- Yes
- No

Please let us know any comments you might have:

_______________________________________________________________

**S2: Study 2: Demographics and identification questionnaire**

I still thought of myself as a parent during the second writing task (CG only)

o Strongly disagree

o Disagree

o Somewhat disagree

o neither agree nor disagree

o Somewhat agree

o Agree

o Strongly Agree

I tried very hard to think of strong arguments for the second writing task: (CG only)

o Strongly disagree

o Disagree

o Somewhat disagree

o neither agree nor disagree

o Somewhat agree

o Agree

o Strongly Agree

I tried very hard to think about myself as a parent when completing the second writing

task: (EG only)

o Strongly disagree

o Disagree

o Somewhat disagree

o neither agree nor disagree

o Somewhat agree

o Agree

o Strongly Agree

I found it difficult to keep thinking of myself as a parent during the second writing task (EG only)

o Strongly disagree

o Disagree

o Somewhat disagree

o neither agree nor disagree

o Somewhat agree

o Agree

o Strongly Agree

**Please indicate how strongly you agree or disagree with the following statements:**

|  | Do not agree at all | |  |  |  | Agree Completely | |
| --- | --- | --- | --- | --- | --- | --- | --- |
| I see myself as a parent |  |  |  |  |  |  |  |
| I am pleased to be a parent |  |  |  |  |  |  |  |
| I feel strong ties with other parents: |  |  |  |  |  |  |  |
| I identify with other parents |  |  |  |  |  |  |  |
| Being a parent is an important reflection of who I am |  |  |  |  |  |  |  |

|  | Do not agree at all | |  |  |  | Agree Completely | |
| --- | --- | --- | --- | --- | --- | --- | --- |
| I see myself as a feminist |  |  |  |  |  |  |  |
| I am pleased to be a feminist |  |  |  |  |  |  |  |
| I feel strong ties with other feminists |  |  |  |  |  |  |  |
| I identify with other feminists |  |  |  |  |  |  |  |
| Being a feminist is an important reflection of who I am |  |  |  |  |  |  |  |

|  | Do not agree at all | |  |  |  | Agree Completely | |
| --- | --- | --- | --- | --- | --- | --- | --- |
| I feel conflicted between being a parent and being a feminist |  |  |  |  |  |  |  |

**Almost done, please answer a few short questions about yourself:**

How old are you? ________________

What is your first language?

- English is my first language
- Other (please specify) ____
- I am bi-/multilingual (please specify the 2 languages you are most proficient in) _______

Which term best describes your gender?

- Male
- Female
- Non-binary
- Other (please specify) _______

Please indicate your nationality _____________________

How old is your youngest child? _______

Do you currently have children living with you?

- Yes
- No

Please let us know any comments you might have:

_______________________________________________________________
